# Development of a Quick and Highly Sensitive Amplified Luminescent Proximity Homogeneous Assay for Detection of Saxitoxin in Shellfish

**DOI:** 10.3390/toxins16080341

**Published:** 2024-08-02

**Authors:** Chenhao Zhao, Zhi Zhang, Jiayu Li, Yaofan Lu, Fuyuan Ma, Zheng Wang, Jiaxin Geng, Biao Huang, Yuan Qin

**Affiliations:** College of Life Sciences and Medicine, Zhejiang Sci-Tech University, Hangzhou 310018, China; zhaochenhao202408@163.com (C.Z.); zhangzhiwyyx@163.com (Z.Z.); jiayuli2021@163.com (J.L.); luyaofan2023@163.com (Y.L.); wangzheng20223090@163.com (Z.W.); gengjiaxin0923@163.com (J.G.)

**Keywords:** saxitoxin, immunoassay, amplified luminescent proximity homogeneous assay, biotoxin, paralytic shellfish poisoning, shellfish

## Abstract

Saxitoxin (STX), an exceptionally potent marine toxin for which no antidote is currently available, is produced by methanogens and cyanobacteria. This poses a significant threat to both shellfish aquaculture and human health. Consequently, the development of a rapid, highly sensitive STX detection method is of great significance. The objective of this research is to create a novel approach for identifying STX. Therefore, amplified luminescent proximity homogeneous assay (AlphaLISA) was established using a direct competition method based on the principles of fluorescence resonance energy transfer and antigen–antibody specific binding. This method is sensitive, rapid, performed without washing, easy to operate, and can detect 8–128 ng/mL of STX in only 10 min. The limit of detection achieved by this method is as low as 4.29 ng/mL with coefficients of variation for the intra-batch and inter-batch analyses ranging from 2.61% to 3.63% and from 7.67% to 8.30%, respectively. In conclusion, our study successfully establishes a simple yet sensitive, rapid, and accurate AlphaLISA method for the detection of STX which holds great potential in advancing research on marine biotoxins.

## 1. Introduction

In recent decades, human activities have caused environmental pollution that has altered the quality of water resources, leading to the rapid proliferation and accumulation of bacteria and algae which are resistant to traditional removal methods, ultimately leading to the emergence of red tides [1,2]. The production of toxins by certain red tide organisms has had a profound impact on the aquaculture and fishing industries, posing an even greater challenge to the preservation of marine ecosystems [1,3]. Based on the different symptoms of intoxication, marine biotoxins can be categorized into the following four main groups: paralytic shellfish toxins (PSTs), diarrhetic shellfish toxins, amnesic shellfish toxins, and neurotoxic shellfish toxins [4]. PSTs are naturally produced by certain species of cyanobacteria (*Anabaena* sp., some *Aphanizomenon* spp., *Planktothrix* sp., *Cylindrospermopsis* sp., *Lyngbya* sp.) and marine dinoflagellates (*Alexandrium* sp., *Gymnodinium* sp., *Pyrodinium* sp.) [5].

Saxitoxin (STX) is classified as one of the most potent carbamates among PSTs, extensively studied in marine environments due to its high toxicity. The molecular weight of STX is 299 g/mol and C_10_H_7_N_7_O_4_ is the molecular structure of STX. More than 50 natural analogs of STX, such as neosaxitoxin (neoSTX), decarbamoylsaxitoxin (dcSTX), C-toxin, gonyautoxins (GTX), and derivatives, have been discovered to date, and many of these natural analogs are also structurally similar to STX. Meanwhile STX Meanwhile STX exhibits excellent solubility in water and remarkable stability under both thermal and acidic conditions [4,6]. STX is taken up by shellfish through non-selective filtration and undergoes transformation and accumulation in their digestive glands. Subsequently, it can be transferred to humans via the food chain, posing a threat to human health and safety [7,8]. The domestic cooking and steaming methods are generally inadequate for the inactivation of STX. Consequently, inadvertent ingestion of shellfish contaminated with STX can lead to the blockade of Na^+^ channels by STX, resulting in the inhibition of nerve impulse transmissions. These physiological alterations may subsequently manifest as symptoms including numbness, headaches, tingling sensations, muscle weakness, respiratory failure, and even fatality [9,10,11]. In addition, STX is one of the shellfish toxins that has been identified as widespread in marine waters, relatively more toxic and highly hazardous to human health. Previous studies have indicated that many countries and coastal areas such as Malaysia, Venezuela, Tasmania, and India recurrently suffer from shellfish contamination by PSTs [12]. Moreover, in coastal areas of China, outbreaks of paralytic shellfish poisonings have occurred after the consumption of toxic bivalves, which not only risks human safety, but also hinders local economic development [13]. To mitigate the impact of STX on human society, the maximum regulatory limit of STX is as low as 800 μg·kg^−1^ in the EU [14]. Consequently, there is an urgent demand to establish an effective method for the detection of STX to preserve the safety of the people and minimize the economic losses.

With the deepening of shellfish research, corresponding detection techniques have been developed, including mouse bioassay (MBA) [15], high performance liquid chromatography (HPLC) [16], liquid chromatography–mass spectrometry (LC-MS/MS) [17], and immunoassay. The application of MBA is simple and universal, but the technique is unsuitable for on-site detection, characterized by low sensitivity, specificity, and reproducibility [18]. HPLC and LC-MS/MS allow for selective identification, separation, and sensitive quantification to be performed [8]. Immunoassay methods, which utilize antigen–antibody specific binding to detect analytes, are characterized by simple extraction and detection, fast execution, and without the need for high-tech instruments and high-quality testing personnel, having been applied to the detection of various types of marine toxins [19]. However, currently, according to prevalent ethics standards, MBA has been gradually replaced by various instrumental procedures such as HPLC and LC-MS/MS (although they often are complicated to run and require experts) [20]. For immunoassay, ELISA is susceptible to enzymatic interference, is sensitive to environmental factors of temperature and pH, and has the drawbacks of poor specificity and high cross-reactivity [21]. However, commercial ELISA test kits are available for many toxins, although some these kits are not 100% specific for all toxins. Lateral flow immunoassay is not capable of accurate, quantitative determination [22]. Immunosensors like surface plasmon resonance are usually limited by their sensitivity and matrix interference [23]. Radioimmunoassay is hindered by issues associated with the disposal of radioactive laboratory waste [24]. Therefore, the establishment of a novel STX detection method with high sensitivity, convenient operation, no cleaning, and short detection time is of significant application value.

AlphaLISA is a bead-based luminescent oxygen channel that utilizes Förster resonance energy transfer. This assay employs photosensitive microspheres and luminescent microspheres as carriers. When the antigen-coated photosensitive microspheres are excited by a 680 nm laser, singlet oxygen is released with a half-life of 4 μs and limited diffusion distance in an aqueous solution to approximately 200 nm [25,26]. After specific binding of the antigen–antibody, resulting in a distance of less than 200 nm between the photosensitive microspheres and the luminescent microspheres coated with antibodies, an amplified fluorescent signal is generated within the luminescent microspheres, leading to light emissions at 615 nm [27,28]. AlphaLISA is characterized by high sensitivity and specificity, a rapid reaction time, low background noise, and simple experimental procedure [29]. Compared to other immunoassays, this method demonstrates relatively low background signal due to the shorter lifetime of single-linear oxygen in solution. Additionally, it offers reduced detection time owing to its homogeneous nature which eliminates the need for washing steps [26]. In recent years, AlphaLISA has witnessed a gradual adoption in the detection of diverse small molecule compounds and protein macromolecules, kinase analysis, as well as protein–protein interactions, encompassing applications in the domains of medicine and food [30,31]. However, there is a paucity of literature reports on the utilization of AlphaLISA for marine toxin detection. In our previous investigation, we successfully established an AlphaLISA assay for okadaic acid (OA), capitalizing on its non-wash properties and significantly reducing the assay time to one-fourth compared to ELISA. Furthermore, we optimized both the detection range and assay sensitivity [32]. Therefore, the objective of this study was to establish a novel, rapid, and accurate AlphaLISA assay for STX, contributing to enhancing marine toxin detection capabilities.

## 2. Results

### 2.1. Detection Principle of STX-AlphaLISA

**Figure 1:**
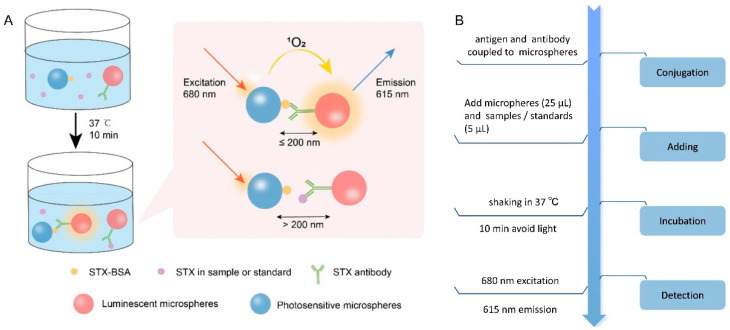
(**A**) AlphaLISA System Schematic for STX detection; (**B**) the flowchart of STX-AlphaLISA.

In this study, a competitive approach was utilized, where the AlphaLISA luminescent microspheres coupled with STX-BSA were in competition with the STX present in the sample or standards for binding sites on AlphaLISA photosensitive microspheres conjugated to the STX antibody. Upon binding of the STX-BSA with photosensitive microspheres to the antibodies coupled with luminescent microspheres, the distance between these two types of microspheres decreased to less than 200 nm due to specific antigen–antibody interactions. This proximity induced the decomposition of oxygen molecules in the surrounding environment under excitation light irradiation, enabling the energy transfer to europium atoms on the luminescent microspheres and subsequent fluorescence generation. On the contrary, no fluorescence was generated when the antibody interacted with STX from standards or samples. The detection principle is depicted in Figure 1A, and Figure 1B illustrates the experimental flow. Initially, 25 μL STX-BSA-coupled photosensitive microspheres were introduced into a 96-well plate, followed by the addition of 25 μL luminescent microspheres conjugated with STX antibodies subsequent to the introduction of 5 μL of either shellfish samples or STX standards. Finally, shaking and incubating the plate at 37 °C for 10 min away from light promoted antigen–antibody binding.

### 2.2. Optimization of STX-AlphaLISA

**Figure 2:**
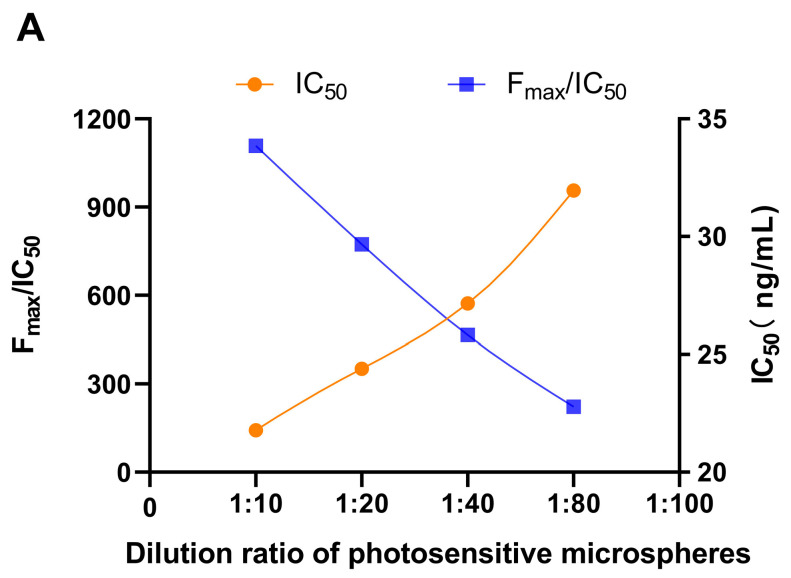

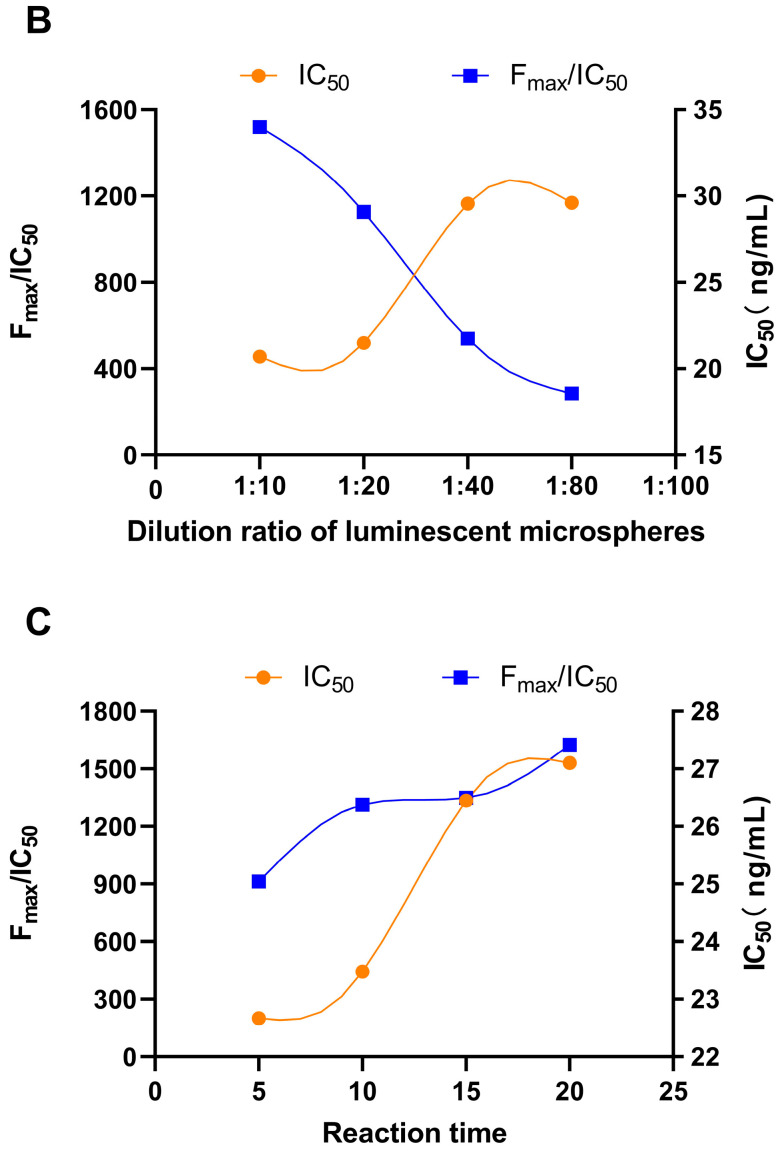
Optimization conditions of STX-AlphaLISA. (**A**) F_max_/IC_50_ and IC_50_ values in 1:10, 1:20, 1:40, and 1:80 dilution ratios of STX-BSA-coupled photosensitive microspheres; (**B**) F_max_/IC_50_ and IC_50_ values in 1:10, 1:20, 1:40, and 1:80 dilution ratios of antibody-coupled luminescent microspheres; (**C**) F_max_/IC_50_ and IC_50_ values in 5, 10, 15, or 20 min reaction times.

To achieve an optimal assay detection performance, the experimental conditions for photosensitive and luminescent microspheres were optimized based on a smaller IC_50_ value and higher F_max_/IC_50_ ratio. The optimal experimental conditions were selected using the controlled variable method. Additionally, STX standard solutions of 0, 8, 16, 32, 64, and 128 ng/mL were prepared according to the concentrations of the samples extracted in the experiments for the construction of the standard curves. Initially, the dilution ratios of STX-BSA coupled photosensitive microspheres were optimized through screening. Four gradient dilution ratios of 1:10, 1:20, 1:40, and 1:80 of STX-BSA-coupled photosensitive microspheres were selected for optimal screening. Meanwhile, luminescent microspheres coupled with STX antibodies were diluted at a ratio of 1:10, and the oscillation reaction was consistently conducted for 10 min. The results depicted in Figure 2A demonstrate lower IC_50_ values and higher F_max_/IC_50_ values when utilizing STX-BSA-coupled photoreceptor microspheres at a dilution ratio of 1:10. The optimization of luminescent microspheres, conjugated with STX antibodies, was performed by employing the determined 1:10 dilution ratio of STX-BSA-coupled photosensitive microspheres. The duration of response was 10 min, while the dilution ratios varied at 1:10, 1:20, 1:40, and 1:80. Figure 2B indicates that the IC_50_ value is smaller and the F_max_/IC_50_ ratio is higher for the dilution ratio of 1:10. The optimal reaction time was determined to be either 5, 10, 15, or 20 min based on the optimal microsphere dilution ratio of 1:10. Figure 2C illustrates that under a reaction time of only 10 min, the IC_50_ value decreases while the F_max_/IC_50_ increases. The present study investigated the optimal parameters for achieving a dilution ratio of 1:10 for both photosensitive microspheres and luminescent microspheres, as well as determining an ideal reaction duration of 10 min.

### 2.3. Identification of STX-AlphaLISA

**Figure 3:**
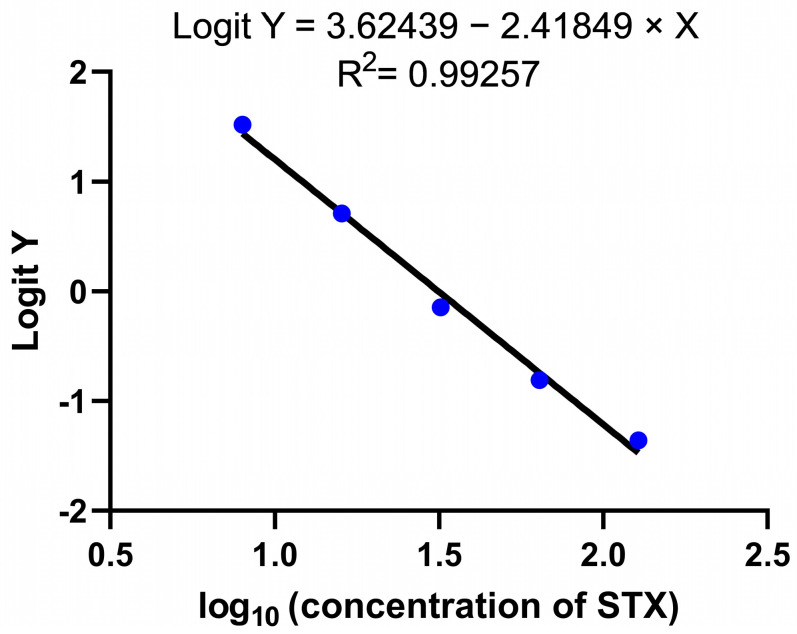
STX-AlphaLISA standard curve.

The performance of the STX-AlphaLISA assay was evaluated through various assessments, including sensitivity, precision, specificity, and recovery. The standard curve of the STX-AlphaLISA was established according to the optimal experimental conditions. The standard curve equation illustrated in Figure 3 is Logit Y = 3.62439 − 2.41849 × X, R^2^ = 0.99257, where Logit Y = ln (y/1 − y), y = F/F_0_, and X = lg (concentration of STX). The findings unveiled that the LOD was 4.29 ng/mL (107.25 μg/kg), while the LOQ was 13.97 ng/mL (349.25 μg/kg) within the detection linear range of 8–128 ng/mL. The coefficients of variation were 2.61–3.63% versus 7.67–8.30% for the intra- and inter-assay batches, respectively, as shown in Table 1. MC, AF, OA, and DTX-1 were detected based on the established AlphaLISA method to determine the cross-reactivity rate. The cross-reactivity rates of MC, AF, OA, and DTX-1 were all found to be less than 1% according to the data presented in Table 2. This indicates the absence of any significant occurrence of cross-reactivity. The recoveries of the established AlphaLISA assay for shellfish samples, ranging from 91.51% to 111.13%, are summarized in Table 3, indicating a high level of accuracy achieved by the developed method.

### 2.4. Application of STX-AlphaLISA

**Figure 4:**
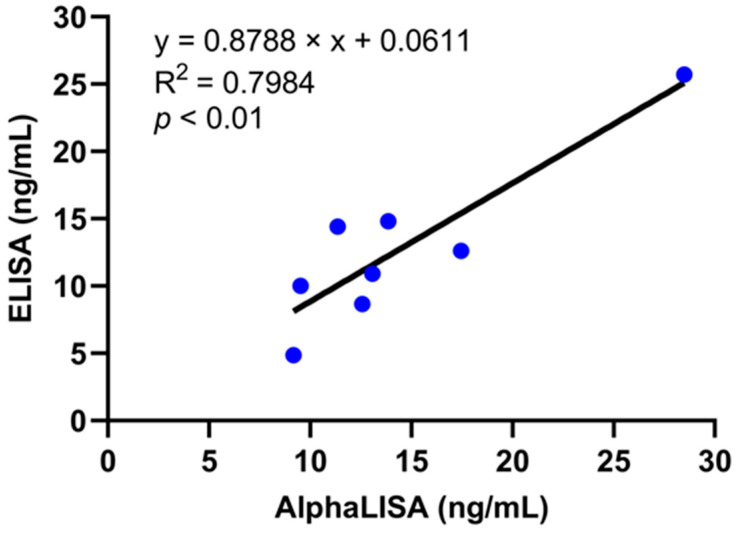
Comparison of AlphaLISA and ELISA sample results.

Subsequently, the STX-AlphaLISA was employed for the detection of STX in actual samples. The oyster and mussel samples were supplemented with STX and analyzed using the STX-AlphaLISA method, while the results were confirmed by comparison with ELISA analysis. The correlation curves presented in Figure 4 demonstrate a significant correlation (*p* < 0.01). The successful application of the STX-AlphaLISA developed in this research was demonstrated for the identification of shellfish samples, and its performance was compared to widely employed ELISA techniques. Our results showed some correlation with the ELISA results, where the R^2^ was 0.7984. Since the concentration of the samples to be tested is close to the LOD of both methods at lower concentrations, it makes the results of the samples at lower concentrations subject to some error.

## 3. Discussion

STX is a prominent subject of study, primarily synthesized by dinoflagellates and cyanobacteria, exhibiting a global distribution. Currently, an effective antidote for STX remains elusive, despite its recognition as one of the most potent naturally occurring neurotoxins [33]. STX is transferred across the food chain to various aquatic organisms, including humans, exerting detrimental effects on human health and posing a significant threat to the global shellfish industry. Among aquatic organisms, bivalves (mussels, oysters, scallops, clams) are more capable of accumulating PSTs, and of these, mussels have become a traditional bioindicator species for assessing environmental quality due to their high tolerance and ability to accumulate PSTs [34]. Meanwhile, due to their prevalence as consumable seafood and the significant implications for food safety, oysters and mussels were chosen as the focal species in this study on STX detection. Given the restricted access to natural shellfish samples abundant in shellfish toxins due to stringent regulatory measures, in this study, we opted for the artificial addition of shellfish toxins. Furthermore, all samples were simultaneously subjected to ELISA methods, widely employed in contemporary research, to validate the precision of experimental outcomes.

The present study established an AlphaLISA assay for STX based on a direct competition method, which is a highly sensitive homogeneous photoexcitation chemiluminescence immunoassay with minimal background signal, elimination of cleaning and separation steps, negligible matrix interference, and requirement of only a small sample volume for testing. In previous studies, AlphaLISA has demonstrated excellent sensitivity, specificity, and reproducibility for the detection of botulinum toxin type A in complex sample matrices within a wide dynamic range of 0.05 ng/mL to 500 ng/mL, exhibiting robust performance regardless of acidity, viscosity, or food matrix composition [35]. Furthermore, an OA-AlphaLISA assay was established in our previous research, with exceptional sensitivity and rapid response times [32]. In this study, the range of the STX-AlphaLISA established was 8–128 ng/mL, which is consistent with the acute reference dose standards of the EFSA and the actual testing requirements for STX. The value of IC_50_ was 31.04 ng/mL, LOD was 4.29 ng/mL, and LOQ was 13.67 ng/mL. The coefficients of variation were 2.61–3.63% and 7.67–8.30% for intra-batch and inter-batch, respectively, and the recoveries ranged from 91.51 to 111.13%. Cross-reactivities with MC, AF, OA, and DTX-1 were less than 1%, which indicated that no significant cross-reactivity was observed and the specificity was good. The successful application of the STX-AlphaLISA developed in this research was demonstrated for the identification of shellfish samples, and its performance was compared to widely employed ELISA techniques. Compared with the ELISA assay, AlphaLISA, as a one-step system, eliminates the cleaning step by utilizing europium-containing microspheres as carriers, greatly shortens the reaction time and reduces the consumption of materials, avoids the use of enzymes so that better stability is achieved, and also mitigates the likelihood of generating false-positive outcomes. Furthermore, AlphaLISA, being a homogeneous assay where the immunoreaction components are dispersed in solution, enhances reaction efficiency and reduces the time required. Meanwhile, due to the limitation of the diffusion range of photosensitive microspheres releasing single-linear oxygen, it is difficult for photosensitive microspheres and luminescent microspheres dispersed in solution to randomly combine luminescence, resulting in low background noise [35]. Consequently, the implementation of STX-AlphaLISA in this study significantly reduces the detection time to a mere 15 min, nearly one-quarter of that of ELISA, on the basis of a no-wash strategy, which had good specificity and low cross-reactivity.

Hitherto, there has been no previous study that has linked the AlphaLISA assay to STX detection, and the STX-AlphaLISA method established in this study provides a new idea for the detection of STX. Compared to MBA, the STX-AlphaLISA offers greater sensitivity and specificity, allowing for quantitative detection and is not harmful to animals [18]. Compared to HPLC and LC-MS/MS, AlphaLISA uses relatively inexpensive and simple instrumentation due to its properties of a homogeneous phase and stronger resistance to matrix interference, with easy operation, and no need for purification of toxins from the sample [36]. The STX-AlphaLISA offers a wider range of reaction temperatures and lower cost compared to immuno-chromatography, making it suitable for diverse environments [37] Compared to the SERS aptamer sensor, STX-AlphaLISA has better stability and simpler experimental operation [38]. Compared with ELISA, the STX-AlphaLISA established in this experiment has good stability, shorter reaction time (10 min), better specificity (less than 1%), and facilitates automation [21]. The sensitivity of this study is good and has satisfied the requirements for the detection of toxins in shellfish samples, which is beneficial for the early detection of shellfish samples, facilitating the quality control of bivalve shellfish and providing early warning of STX outbreaks. Employing the principle of Förster resonance energy transfer, this experiment is designed to produce fluorescence only when the STX antibody binds specifically to STX, realizing a significant reduction in reaction time while maintaining specificity and high sensitivity. With the continuous advances in novel materials, embedding agents, and antibody production technologies, AlphaLISA technology is poised to exhibit enhanced precision and sensitivity across diverse environmental settings.

The experiment is subject to certain limitations. First, the sensitivity of STX-AlphaLISA is low compared to instrumental correlation methods such as HPLC, which is insufficient for the detection of STX samples in all environments. Secondly, due to the small sample size of the assay, no natural shellfish samples were tested for STX. Thirdly, there is no comparison of the concentrations measured with AlphaLISA to concentrations measured by instrumental methods, such as MS or HPLC. Finally, no PST analogues such as neoSTX, dcSTX, GTX1&4, and GTX2&3 were tested in this assay. In future investigations, we will continue to optimize the sensitivity of the STX-AlphaLISA and collect shellfish samples containing naturally occurring STX and STX-containing water bodies to enhance this detection. We will also further elucidate the cross-reactivity of the AlphaLISA method to PST analogs.

## 4. Conclusions

This study has successfully established the STX-AlphaLISA method, which is characterized by its simplicity, rapidity, accurate results, and high specificity. This innovative approach provides a novel perspective for the detection of STX and holds great potential for automation and high-throughput screening. Moreover, it can be effectively applied to the early detection of bivalve shellfish contamination, thereby offering promising prospects in marine biotoxin detection. The method effectively balances the sensitivity of detection and operational convenience, thereby presenting a valuable application of immunoassay methods in the field of shellfish toxin detection. In the future, STX-containing shellfish will be utilized for on-site detection, while optimizing the conditions to enhance the specificity that especially focus on PST analogs and stability of the test kit. This advance in testing is anticipated to find applications in seafood aquaculture, environmental testing, and food safety domains. Furthermore, we aim to explore the feasibility of employing the AlphaLISA method for detecting additional shellfish toxins.

## 5. Materials and Methods

### 5.1. Reagents and Instruments

STX-bovine serum albumin (BSA), STX standard, STX mouse monoclonal antibody, and STX ELISA kits were procured from Beosen Food Safety Technology (Wuxi, China). The AlphaLISA luminescent and photoreceptor microspheres were provided by Weidu Biological Technology (Suzhou, China). Tris-base and MES were bought from Seebio Biotechnology (Shanghai, China). BSA and Proclin 300 were obtained from Sigma-Aldrich (Saint Louis, USA). Microcystin (MC) and aflatoxin (AF) were purchased from Beosen Food Safety Technology (Wuxi, China). Okadaic acid (OA), dinophysistoxins-1 (DTX-1), and oyster and mussel samples were purchased from the National Marine Environment Monitoring Center of China. Blocking buffer (0.6% Tris-base, 0.2% BSA, 0.9% NaCl, 0.05% Proclin 300), assay buffer (50 mmol/L Tris-HCl, 0.01% Tween-20, 0.05% NaN_3_, 0.2% BSA, 20 µM DTPA, 0.9% NaCl, pH 7.8), labeling buffer (50 mM MES, pH 6.0), and standard working solution (2.5 mmol/L Tris-HCl, 0.05% Proclin 300, 0.5% BSA) were prepared in-house by our laboratory.

Ultracel-50k ultrafiltration tubes, procured from Millipore (Burlington, MA, USA), were used to exchange STX monoclonal antibodies and STX-BSA buffers. For complete dispersion of the microspheres, an ultrasonic cleaner purchased from Suntad (Guangdong, China) was used. Reaction vessels were 96-well plates obtained from Sigma-Aldrich (Saint Louis, MO, USA). Immune responses were stimulated with a micro-oscillator and an electric heating incubator. The fluorescence values of the AlphaLISA reaction were measured using by a photoluminescence detector purchased from Boyang Medical Instruments (Shanghai, China).

### 5.2. Conjugated Microspheres

The photosensitive microspheres were conjugated with STX-BSA, while the luminescent microspheres were conjugated with STX monoclonal antibodies. Then, the filtration membranes, pre-buckled to the wall of the ultrafiltration tube, were supplemented with both STX-BSA and STX antibodies. The resulting mixture was then centrifuged at 10,000 RPM (9600× *g*) for 5 min. Subsequently, 300 μL of MES was added to the ultrafiltration tubes and centrifuged at 10,000 RPM (9600× *g*) for 5 min. This process was repeated 8 times with the filtrate being removed every two cycles. The 50 μL MES solution was added to the ultrafiltration tube and stood for 2 min after the last centrifugation. After inverting the tubes, the centrifugation process was employed to collect the filtrate at a speed of 3000 RPM (900× *g*) for a duration of 1 min. Subsequently, the photosensitive and luminescent microspheres were transferred into a centrifuge tube, followed by the addition of 900 μL of labeling buffer. The mixture was then subjected to centrifugation at 13,000 RPM (16,200× *g*) for a duration of 10 min, followed by discarding the supernatant. The microspheres were dispersed by adding 1 mL of labeling buffer and sonicating for 15 min. A labeling buffer was utilized to prepare a solution containing N-Hydroxy succinimide (NHS) and 1-(3-Dimethylaminopropyl)-3-ethylcarbodiimide (EDC) at a concentration of 20 mg/mL. Subsequently, the cleaned microspheres were treated with 10 μL of NHS solution followed by the addition of 5 μL of EDC solution with vigorous mixing. The mixed solution was incubated for 20 min at 37 °C in the absence of light, then centrifuged at 13,000 RPM (16,200× *g*) for 10 min removing the supernatant. The microspheres were resuspended with 100 μL of labeling buffer, then sonicated until obvious aggregation of microspheres disappeared. Repeating the aforementioned procedure twice, STX antibodies and STX-BSA were introduced into photosensitive microspheres and photosensitized microspheres, respectively, with rapid mixing. The mixture was incubated for 2 h at 37 °C away from light. When the incubation time ended, 100 μL of sealing solution was added and the mixture was further incubated for 1 h at 37 °C away from light. Subsequently, the mixture was centrifuged at 13,000 RPM (16,200× *g*) for 10 min to remove the supernatant. It was then resuspended with 1 mL of working dilution and sonicated for dispersion. Following two repetitions, a microsphere solution with a concentration of 1 mg/mL was prepared and stored protected from light.

### 5.3. Optimization

For enhancing detection effectuality, the reaction time and the dilution of photosensitive and luminescent microspheres were optimized through selecting the lower IC_50_ values (half of the maximum inhibitory concentration) and higher F_max_/IC_50_ values (F: fluorescence measurement; F_max_: the fluorescence measurement at maximum fluorescence value—0 concentration).

### 5.4. Method Evaluation

Sensitivity is defined as the concentration corresponding to the mean fluorescence value at 0 concentration which requires 10 repeated determinations deducting doubled standard deviation (SD). According to the American Chemical Society Committee on Environmental Improvement, the limit of detection (LOD) and limit of quantitation (LOQ) correspond to three times (3 × SD) and ten times (10 × SD), respectively [39].

Precision refers to the approximation between repeated measurements of a given sample under the identical conditions. Intra-assay coefficients of variation are calculated through the formula where SD is divided by the average concentration values measured from identical samples repeatedly three times. The coefficients of variation between batches are calculated by SD division of the mean concentration of three repeated experiments.

Under optimal reaction conditions, the cross-reactivity rates of other marine biotoxins, including MC, AF, OA, and DTX-1, are inversely proportional to their specificity.

The recovery rates are defined to assess the accuracy of AlphaLISA, a method used for quantitative analysis. The determined STX standard was added to the sample in order to determine its concentration. Recovery can be figured out by dividing the real concentration by the theoretical concentration and multiplying by 100%.

### 5.5. Sample Processing

The oyster and mussel samples were weighed at 20 g with a precision of 0.1 g, and then 50 mL of hydrochloric acid solution (0.1 mol/L) was added. Boil and stir for 5 min. Then, it should be centrifuged at 6000 RPM (3500× *g*) for 10 min at 4℃ and adjust the pH of the supernatant below 4.0 with hydrochloric acid solution (5 mol/L). The extract was mixed well with 900 μL of PBS solution (pH 7.4) after adding 100 μL. STX was artificially incorporated into shellfish samples.

### 5.6. ELISA Detection Protocol for STX

The ELISA method was utilized to detect the STX sample to further verify the reliability of the established AlphaLISA protocol. According to the instructions of the ELISA kit, STX standard solution at concentrations of 0, 8, 16, 32, 64, and 128 ng/mL and the solution to be tested were added. The enzyme labeled antibody solution was added in the amount of 50 μL to react for 30 min, then 100 μL of color development solution was added, and the termination solution was added after 10 min. Finally, the optical density (OD) value was read in the enzyme labeling instrument. The OD values measured from 1–6 wells of gradient STX standard solutions (0, 8, 16, 32, 64, 128 ng/mL) were plotted as a standard curve through logit-log linear regression model. The concentration corresponding to the measured sample was determined based on the standard curve using its OD value.

### 5.7. Statistical Analysis

The tests were repeated three times in order to ensure accuracy and reliability. The AlphaLISA results were compared with the ELISA method to analyze their correlation, while IC_50_ values were determined using IBM SPSS Statistics 26 (SPSS Inc., Chicago, IL, USA). Either Pearson’s correlation test or Spearman’s correlation test was used to perform the normality test. A significance level of *p* < 0.05 was considered statistically significant.

## Figures and Tables

**Table 1:** Precision of STX-AlphaLISA.

	Samples	Average Concentration of STX (ng/mL)	Standard Deviation	Variable Coefficient/(%)
intra-assay	oyster	46.51	1.69	3.63
	mussel	28.57	0.75	2.61
inter-assay	oyster	42.43	3.52	8.30
	mussel	31.03	2.38	7.67

**Table 2:** Cross-reactivity of AlphaLISA with other marine toxins.

Toxin	Cross-Reactivity (100%)
MC	0.96
AF	0.25
OA	0.75
DTX-1	0.57

**Table 3:** Recovery rate of samples spiked with STX-AlphaLISA.

Sample	Spiked Concentration (ng/mL)	AlphaLISA	
		Mean ± SD (%)	RSD (%)
1	10	91.51 ± 5.28	5.77
2	50	111.13 ± 8.69	7.82
3	100	102.82 ± 6.56	6.38

RSD: relative standard deviation.

## Data Availability

The data presented in this study are available in this article.

## References

[1] Chorus I., Welker M. (2021). Toxic Cyanobacteria in Water.

[2] Zhao X., Zeng S., Feng H., Wang Y., Li S., Zhou X., Wang M., Rei L. (2022). Antifouling performance of in situ synthesized chitosan-zinc oxide hydrogel film against alga *M. aeruginosa*. Int. J. Biol. Macromol..

[3] Munoz M., Nieto-Sandoval J., Cirés S., de Pedro Z.M., Quesada A., Casas J.A. (2019). Degradation of widespread cyanotoxins with high impact in drinking water (microcystins, cylindrospermopsin, anatoxin-a and saxitoxin) by CWPO. Water Res..

[4] Serrano P.C., Nunes G.E., Avila L.B., Reis C.P.S., Gomes A.M.C., Reis F.T., Sartorelli M.L., Melegari S.P., Matias W.G., Bechtold I.H. (2021). Electrochemical impedance biosensor for detection of saxitoxin in aqueous solution. Anal. Bioanal. Chem..

[5] Bratakou S., Nikoleli G.P., Siontorou C.G., Nikolelis D.P., Karapetis S., Tzamtzis N. (2017). Development of an Electrochemical Biosensor for the Rapid Detection of Saxitoxin Based on Air Stable Lipid Films with Incorporated Anti-STX Using Graphene Electrodes. Electroanalysis.

[6] Hou L., Jiang L., Song Y., Ding Y., Zhang J., Wu X., Tang D. (2016). Amperometric aptasensor for saxitoxin using a gold electrode modified with carbon nanotubes on a self-assembled monolayer, and methylene blue as an electrochemical indicator probe. Microchim. Acta.

[7] Cheng S., Zheng B., Yao D., Kuai S., Tian J., Liang H., Ding Y. (2018). Study of the binding way between saxitoxin and its aptamer and a fluorescent aptasensor for detection of saxitoxin. Spectrochim. Acta Part A Mol. Biomol. Spectrosc..

[8] Gao S., Zheng X., Wu J. (2017). A biolayer interferometry-based competitive biosensor for rapid and sensitive detection of saxitoxin. Sens. Actuators B Chem..

[9] O’Neill K., Musgrave I.F., Humpage A. (2016). Low dose extended exposure to saxitoxin and its potential neurodevelopmental effects: A review. Environ. Toxicol. Pharmacol..

[10] Woo C.K., Bahna S.L. (2011). Not all shellfish “allergy” is allergy!. Clin. Transl. Allergy.

[11] Zhang W., Dixon M.B., Saint C., Teng K.S., Furumai H. (2018). Electrochemical Biosensing of Algal Toxins in Water: The Current State-of-the-Art. ACS Sens..

[12] Weng Q., Zhang R., Wu P., Chen J., Pan X., Zhao D., Wang J., Zhang H., Qi X., Wu X. (2023). An Occurrence and Exposure Assessment of Paralytic Shellfish Toxins from Shellfish in Zhejiang Province, China. Toxins.

[13] Zheng R., Yang Y., Zhang W., Hua Y. (2023). Contamination status of paralytic shellfish toxins in shellfish from Southeastern China in 2017–2021. Environ. Sci. Pollut. Res. Int..

[14] Gu H.J., Hao L.L., Ye H., Ma P.F., Wang Z.P. (2021). Nuclease-assisted target recycling signal amplification strategy for graphene quantum dot-based fluorescent detection of marine biotoxins. Microchim. Acta.

[15] Cheng J.P., Pi S.S., Ye S.F., Gao H.M., Yao L., Jiang Z.Y., Song Y.L., Xi L. (2012). A new simple screening method for the detection of paralytic shellfish poisoning toxins. Chin. J. Oceanol. Limnol..

[16] Podduturi R., Schlüter L., Liu T.T., Osti J.A.S., Moraes M.D.B., Jorgensen N.O.G. (2021). Monitoring of saxitoxin production in lakes in Denmark by molecular, chromatographic and microscopic approaches. Harmful Algae.

[17] Coleman R., Lemire S.W., Bragg W., Garrett A., Ojeda-Torres G., Wharton R., Hamelin E., Thomas J., Johnson R.C. (2017). Development and validation of a high-throughput online solid-phase extraction-liquid chromatography-tandem mass spectrometry method for the detection of gonyautoxins 1&4 and gonyautoxins 2&3 in human urine. Biomed. Chromatogr. BMC.

[18] Handy S.M., Yakes B.J., DeGrasse J.A., Campbell K., Elliott C.T., Kanyuck K.M., Degrasse S.L. (2013). First report of the use of a saxitoxin-protein conjugate to develop a DNA aptamer to a small molecule toxin. Toxicon.

[19] Ling S., Xiao S., Xie C., Wang R., Zeng L., Wang K., Zhang D., Li X., Wang S. (2018). Preparation of Monoclonal Antibody for Brevetoxin 1 and Development of Ic-ELISA and Colloidal Gold Strip to Detect Brevetoxin 1. Toxins.

[20] Dhanji-Rapkova M., O’Neill A., Maskrey B.H., Coates L., Teixeira Alves M., Kelly R.J., Hatfield R.G., Rowland-Pilgrim S.J., Lewis A.M., Algoet M. (2018). Variability and profiles of lipophilic toxins in bivalves from Great Britain during five and a half years of monitoring: Okadaic acid, dinophysis toxins and pectenotoxins. Harmful Algae.

[21] Eangoor P., Indapurkar A.S., Vakkalanka M.D., Knaack J.S. (2019). Multiplexed ELISA screening assay for nine paralytic shellfish toxins in human plasma. Analyst.

[22] Li J., Persson K.M. (2021). Quick detection method for paralytic shellfish toxins (PSTs) monitoring in freshwater—A review. Chemosphere.

[23] Schulz K., Pöhlmann C., Dietrich R., Märtlbauer E., Elßner T. (2019). Electrochemical Biochip Assays Based on Anti-idiotypic Antibodies for Rapid and Automated On-Site Detection of Low Molecular Weight Toxins. Front. Chem..

[24] Humpage A.R., Magalhaes V.F., Froscio S.M. (2010). Comparison of analytical tools and biological assays for detection of paralytic shellfish poisoning toxins. Anal. Bioanal. Chem..

[25] Zhao J., Lv Q., Liu P., Guo L., Zhang L., Zheng Y., Ming L., Kong D., Jiang H., Jiang Y. (2019). AlphaLISA for detection of staphylococcal enterotoxin B free from interference by protein A. Toxicon.

[26] Yu Z.T., Guan H., Cheung M.K., McHugh W.M., Cornell T.T., Shanley T.P., Kurabayashi K., Fu J. (2015). Rapid, automated, parallel quantitative immunoassays using highly integrated microfluidics and AlphaLISA. Sci. Rep..

[27] Xiang Z., Chen X., Zhou X., Qin Y., Zhao X., Wang Y., Li Q., Huang B. (2022). Development and application of a novel aldehyde nanoparticle-based amplified luminescent proximity homogeneous assay for rapid quantitation of pancreatic stone protein. Clin. Chim. Acta.

[28] Zhang L., Lv Q., Zheng Y., Chen X., Kong D., Huang W., Liu P., Jiang H., Jiang Y. (2021). A rapid and accurate method for screening T-2 toxin in food and feed using competitive AlphaLISA. FEMS Microbiol. Lett..

[29] Zong H., Zhang S., Shang X., Jiang H., Zhao Z., Chen S., Wang X., Wang Y., Jiang Y., Li X. (2023). Development of an AlphaLISA assay for sensitive and accurate detection of influenza B virus. Front. Med..

[30] Armstrong C.M., Ruth L.E., Capobianco J.A., Strobaugh T.P., Rubio F.M., Gehring A.G. (2018). Detection of Shiga Toxin 2 Produced by Escherichia coli in Foods Using a Novel AlphaLISA. Toxins.

[31] Lassabe G., Kramer K., Hammock B.D., González-Sapienza G., González-Techera A. (2018). Noncompetitive Homogeneous Detection of Small Molecules Using Synthetic Nanopeptamer-Based Luminescent Oxygen Channeling. Anal. Chem..

[32] Qin Y., Li J., Kuang J., Shen S., Zhou X., Zhao X., Huang B., Han B. (2023). Okadaic Acid Detection through a Rapid and Sensitive Amplified Luminescent Proximity Homogeneous Assay. Toxins.

[33] Vilariño N., Louzao M.C., Abal P., Cagide E., Carrera C., Vieytes M.R., Botana L.M. (2018). Human Poisoning from Marine Toxins: Unknowns for Optimal Consumer Protection. Toxins.

[34] Wu H.Y., Zhang F., Dong C.F., Zheng G.C., Zhang Z.H., Zhang Y.Y., Tan Z.J. (2022). Variations in the toxicity and condition index of five bivalve species throughout a red tide event caused by Alexandrium catenella: A field study. Environ. Res..

[35] Zhang L., Lv Q., Zheng Y., Gao S., Huang W., Liu P., Kong D., Wang Y., Yu Y., Jiang Y. (2022). Rapid and sensitive detection of botulinum toxin type A in complex sample matrices by AlphaLISA. Front. Public Health.

[36] Watanabe R., Kanamori M., Yoshida H., Okumura Y., Uchida H., Matsushima R., Oikawa H., Suzuki T. (2019). Development of Ultra-Performance Liquid Chromatography with Post-Column Fluorescent Derivatization for the Rapid Detection of Saxitoxin Analogues and Analysis of Bivalve Monitoring Samples. Toxins.

[37] Su K.Q., Qiu X.X., Fang J.R., Zou Q.C., Wang P. (2017). An improved efficient biochemical detection method to marine toxins with a smartphone-based portable system-Bionic e-Eye. Sens. Actuators B-Chem..

[38] Bai X., Gong W., Guo Y., Zhu D., Li X. (2023). Detection of saxitoxin by a SERS aptamer sensor based on enzyme cycle amplification technology. Analyst.

[39] Wippermann D., Zonderman A., Zimmermann T., Pröfrock D. (2023). Determination of technology-critical elements in seafood reference materials by inductively coupled plasma-tandem mass spectrometry. Anal. Bioanal. Chem..

